# The TB burden in East Java, Indonesia, post-COVID-19

**DOI:** 10.5588/ijtldopen.24.0270

**Published:** 2024-08-01

**Authors:** S. Palupi, I. Pambudi, T.T. Pakasi, S. Sulistyo, K.K.K. Htet, V. Chongsuvivatwong

**Affiliations:** ^1^ East Java Provincial Government, Prevention and Disease Control, Surabaya, East Java Province, Indonesia;; ^2^ Prince of Songkla University, Department of Epidemiology, Hat Yai, Songkhla, Thailand;; ^3^ Ministry of Health of the Republic of Indonesia, Directorate of Direct Communicable Disease Prevention and Control, Jakarta, Indonesia;; ^4^ Ministry of Health of the Republic of Indonesia, National Tuberculosis Programme, Jakarta, Indonesia.

**Keywords:** tuberculosis, SARS-CoV-2, MDR-TB, lockdowns

Dear Editor,

The COVID-19 pandemic caused unprecedented disruption to global health systems. One of its critical impacts was a sharp decline in the diagnosis and treatment of TB, a major infectious disease that continues to impose a significant health burden worldwide.^1^ This decline was likely caused by lockdowns, overburdened healthcare facilities, fear of seeking medical attention, and the repurposing of TB resources to address the COVID-19 crisis.^2–5^ Early reports suggest that the pandemic could reverse years of progress made in the fight against TB.^6^ East Java, the second-most populous province in Indonesia (with a population of 40 million), has a high TB burden. Like most reported areas, the province experienced a reduction in TB notification cases during the COVID-19 pandemic.^7–12^ If this reduction were just due to the failure of the health system, we would expect a subsequent rebound. This study aims to investigate the changes in TB trends across population subgroups in East Java during different phases of the COVID-19 pandemic.

We conducted a retrospective study of TB cases in East Java using data from Indonesia’s national electronic surveillance system from 2018 to 2023. All records are de-identified. The Institutional Ethics Committee of the Faculty of Medicine at Prince of Songkla University in Hat Yai, Thailand, granted ethical approval under reference number REC 67-216-18-1.

**Figure 1.**
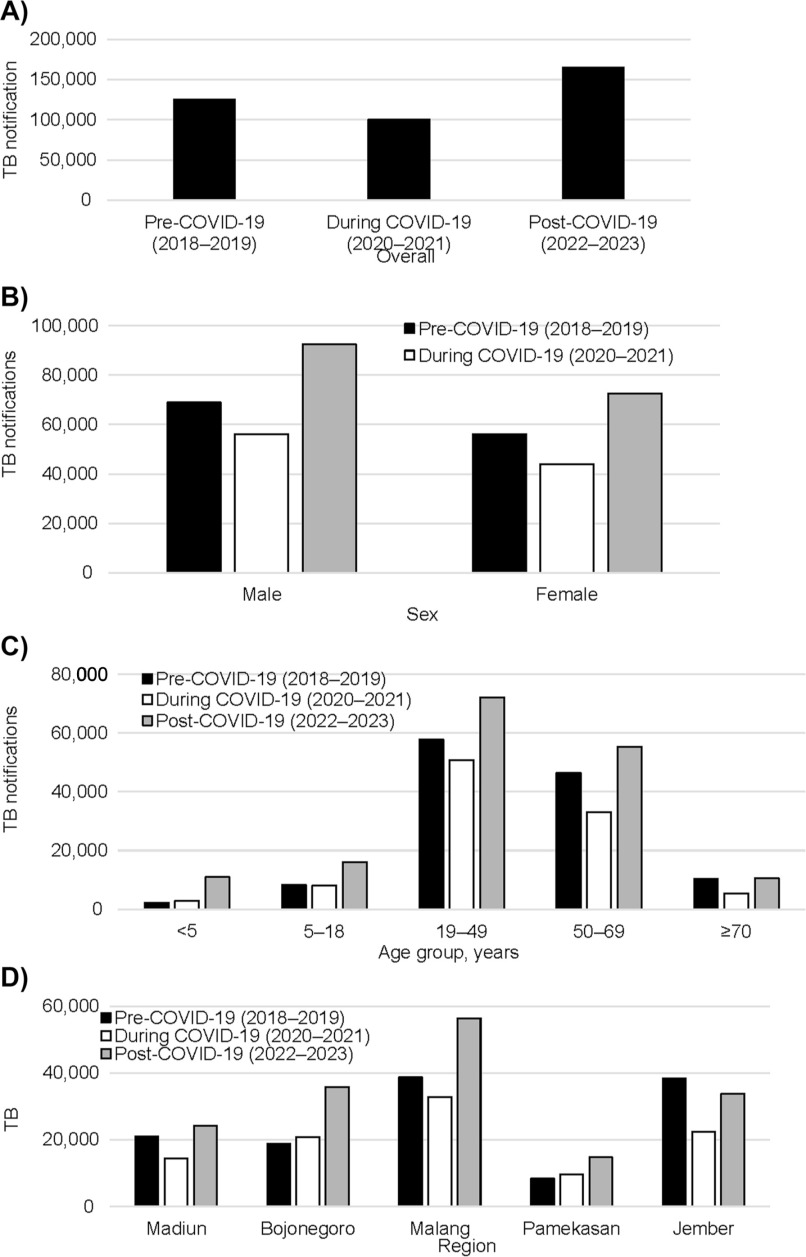
Number of TB notification cases by **A)** overall, **B)** sex, **C)** age group, and **D)** region of East Java pre-, during and post-COVID-19.

**Figure 2.**
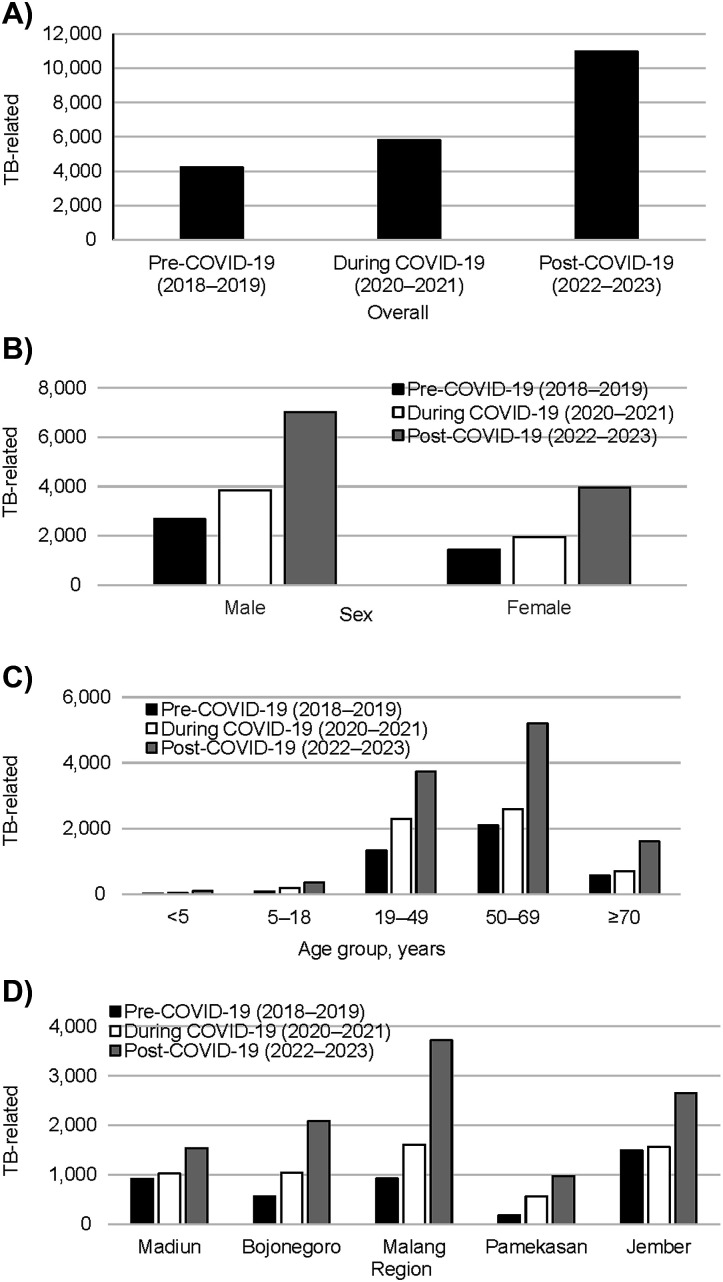
Number of TB-related deaths **A)** overall, **B)** by sex, **C)** by age group, **D)** by region of East Java pre-, during and post-COVID-19.

A summary of the number of TB notification cases across three periods (pre-COVID 2018–2019; during COVID 2020–2021; and post-COVID 2022–2023), classified by sex, age, and region is shown in Figure 1. The percentage changes from the 2018–2019 period are also summarised in the ‘Change’ columns. Overall, TB notifications decreased by 19.9% from 2018-2019 to 2020-2021. However, exceptional increments were seen in children under 5 years old and the cases from Bojonegoro and Pemekasan regions, which showed increases of 30.4%, 11.3%, and 15.9%, respectively. Then, in 2022–2023, TB notification cases rebounded to 31.9% above the pre-COVID-19 period for nearly all subgroups. On the other hand, the summary of TB-related deaths across the three time periods showed no period of decline – see Figure 2. The rates increased by 36.9% in 2020–2021 and surged by 158.9% above baseline in 2022–2023.

There was a temporary decline in TB notifications from 2020–2021, as the healthcare sector was severely disrupted by the COVID-19 pandemic. However, this rebounded in 2022–2023. Overall, our data indicate that the TB burden in Indonesia is increasing. More efforts are, therefore, needed to address the levels of TB transmission and mortality in Indonesia.
